# The Interplay between HGF/c-met Axis and Nox4 in BRAF Mutated Melanoma

**DOI:** 10.3390/ijms22020761

**Published:** 2021-01-13

**Authors:** Francesca Beretti, Francesca Farnetani, Luca Reggiani Bonetti, Luca Fabbiani, Manuela Zavatti, Antonino Maiorana, Giovanni Pellacani, Tullia Maraldi

**Affiliations:** 1 Department of Biomedical, Metabolic and Neural Sciences, University of Modena and Reggio Emilia, 41125 Modena, Italy; francesca.beretti@unimore.it (F.B.); manuela.zavatti@unimore.it (M.Z.); 2 Department of Surgical, Medical, Dental and Morphological Sciences with Interest in Transplant, Oncology and Regenerative Medicine, University of Modena and Reggio Emilia, 41124 Modena, Italy; francesca.farnetani@unimore.it (F.F.); giovanni.pellacani@unimore.it (G.P.); 3 Department of Medical and Surgical Sciences for Mothers, Children and Adults, University of Modena and Reggio Emilia, Azienda Ospedaliero Universitaria Policlinico, 41124 Modena, Italy; luca.reggianibonetti@unimore.it (L.R.B.); luca.fabbiani@unimore.it (L.F.); antonino.maiorana@unimore.it (A.M.)

**Keywords:** melanoma, HGF, oxidative stress, NADPH oxidases

## Abstract

Background: Melanoma is the leading cause of death due to cutaneous malignancy and its incidence is on the rise. Several signaling pathways, including receptor tyrosine kinases, have a role in the development and progression of melanocytic lesions and malignant melanoma. Among those, the hepatocyte growth factor (HGF)/c-met axis is emerging as a critical player because it can play a role in drug resistance. Indeed, 50% of melanoma patients present BRAF mutations, however, all responders develop resistance to the inhibitors typically within one year of treatment. Interestingly, BRAF inhibitors induce reactive oxygen species (ROS) in melanoma cells, therefore, the aim of this study was to investigate a possible interplay between HGF/c-met and ROS sources, such as NADPH oxidases (Nox). Methods: The expression of c-met and Nox were quantified in 60 patients with primary cutaneous melanoma. In vitro experiments on melanoma primary cells and the cell line were performed to dissect the underpinned molecular mechanism. Results: The outcome of interest was the correlation between the high positivity for both Nox4 and c-met and metastasis occurring at least 1 year later than melanoma diagnosis in BRAF mutated patients, in contrast to nonmutated. In vitro experiments demonstrated that the axis HGF/c-met/Nox4/ROS triggers the epithelial-mesenchymal transition. Conclusions: The observed correlation suggests an interplay between c-met and Nox4 in promoting the onset of metastasis. This study suggests that Nox4 inhibitors could be associated to the current therapy used to treat melanoma patients with BRAF mutations.

## 1. Introduction

Hepatocyte growth factor (HGF), a dominant stimulator of epithelial cell division, and its receptor c-met may play an important role in the invasion, motility, proliferation and progression of melanoma. Indeed, c-met overexpression is related to progression and metastatic spread of cutaneous malignant melanomas [1]. Dissimilar to melanocytes, melanoma cells not only express c-met, but also release HGF, thus generating an autocrine loop. Stimulation of the HGF/c-met pathways could be the starting point that promotes several downstream processes that are crucial for melanoma development, such as proliferation, survival, motility, and invasiveness, including distant metastatic niche formation [1].

Moreover, HGF/c-met pathway activation has been demonstrated to elicit both innate and acquired resistance to BRAF inhibitors. BRAF mutations occur in about 50% of melanoma patients. FDA approved BRAF and MEK inhibitors have improved the prognosis of patients with BRAF mutations. However, all responders develop resistance typically within one year of treatment. Recent observations demonstrate that BRAF inhibitors induce reactive oxygen species in melanoma cells [2]. Disruption of redox balance could modulate the effects of BRAF-inhibition in melanoma cells. BRAF-inhibitor resistant melanomas were shown to upregulate NRF2-mediated antioxidant response to maintain cell survival [3].

The ROS/RNS (reactive oxygen and nitrogen species) pool from various sources may form a deleterious feedback circuit for melanomagenesis. Nevertheless, endogenous ROS can act as preventive agents for melanomagenesis as they kill damaged cells, but they can trigger cell proliferation and promote transformation. Thus, the function of ROS in melanomagenesis is indeed complicated, depending on the concentration and localization of ROS production [4].

Intracellular ROS can be generated by the NADPH oxidase family. NADPH oxidase (Nox) activity is induced by UV radiation, and Nox1 protein levels are higher in melanoma cells than in normal melanocytes. Overexpression of Nox1 in melanoma cells is often associated with increased migration/metastasis rate [5,6]. Generally speaking, increased generation of Nox1-derived ROS is functionally required for Ras transformation phenotypes, upregulation of vascular endothelial growth factor, tumor progression and tumor cell migration [7]. Indeed, Ras-transformed cells are highly metastatic, and the Ras oncogene is able to stimulate both matrix metalloproteases production and cell migration [8].

Current evidence suggests that Nox1, Nox4 and Nox5 are expressed in melanocytic lineage [4]. However, while there is no difference in Nox1 expression levels in primary and metastatic melanoma tissues, Nox4 expression is significantly higher in a subset of metastatic melanoma tumors as compared to the primary tumors; suggesting distinct and specific signals and effects for Nox family enzymes in melanoma [6].

It has been reported that Nox4 was up-regulated in more than half of melanoma cell lines tested or that that expression of Nox4 was detected at least in one third of melanoma patients’ samples, suggesting the association of Nox4 expression with some steps of melanoma development [9]. The findings hint that Nox4-generated ROS are required for transformation phenotype of melanoma cells [10].

Nonetheless, it still remains to be examined how Nox4 derived ROS in BRAF mutated melanoma cells regulate their metastatic progression and drug sensitivity. In this study we investigated the first point, starting from a larger view, analyzing Nox4 and c-met expression in early, late and non-metastatic melanoma patients, then focusing on cellular ROS modulation by HGF/c-met and Nox4 inhibition in vitro. The data reported here suggests a link between the HGF/c-met/Nox4 axis and metastatic progression through the epithelial-mesenchymal transition (EMT).

## 2. Results

### 2.1. Study Population and Clinical Follow-Up

In a total of 60 patients, 25 females and 35 males, were included in the study, with a mean age of 57 ± 12 years (see Table 1). Clinical follow-up information was available for a range of 11–72 months. At the time of last clinical follow-up, 6 patients of the MeM (metastatic melanoma) group had died from melanoma, while 54 patients were alive. In the study population, 19 patients did not present recurrences (31.6% NoM, primary melanoma) and 41 patients with recurrences (68.3% MeM). Among MeM group, 9 patients (21.9% MeL) showed only positive lymph nodes, while 16 patients (39.0%) already showed distant metastasis at the time of melanoma diagnosis. Distant metastasis occurred after at least 1 year for 16 MeM patients (39.0%). BRAFV600E mutation was present in 26 patients (52.6% of M and 39.0% of MeM).

### 2.2. Immunohistochemical Analyses Correlated with Malignant Melanoma Type

**Figure 1:**
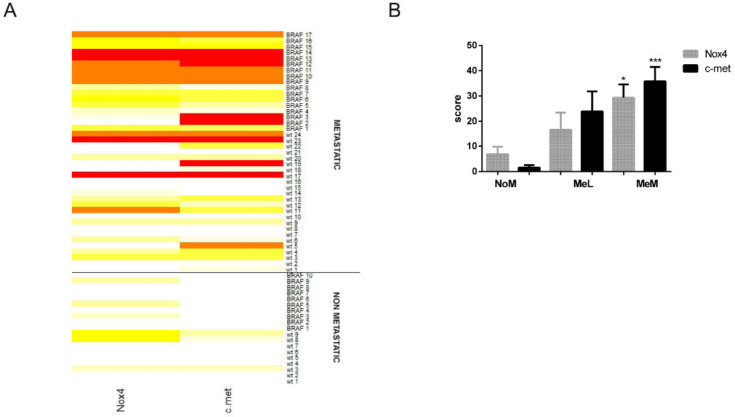
Nox4 and c-met expression in primary melanoma. (**A**) Heat-map showing the Nox4 and c-met IHC staining in primary melanoma, NoM and MeM, WT or with BRAF mutation. White means 0% of positive cells, intense red means 100%, yellow and orange mean levels in between. (**B**) Graph showing immunohistochemical (IHC) evaluation, dividing metastatic melanoma in MeL (positivity only in lymph nodes) and MeM (distant metastasis). * *p-*value < 0.05; *** *p-*value < 0.001.

The immunohistochemical (IHC) results are showed in the heat-map and in the graph of Figure 1A,B. The expression of both Nox4 and c-met was found statistically increasing from M to MeM group, showing a moderate Pearson positive correlation (ρ = 0.685); the MeL group displayed a slight increase on these parameters, compared to NoM group, even if not statistically relevant (Figure 1B).

**Figure 2:**
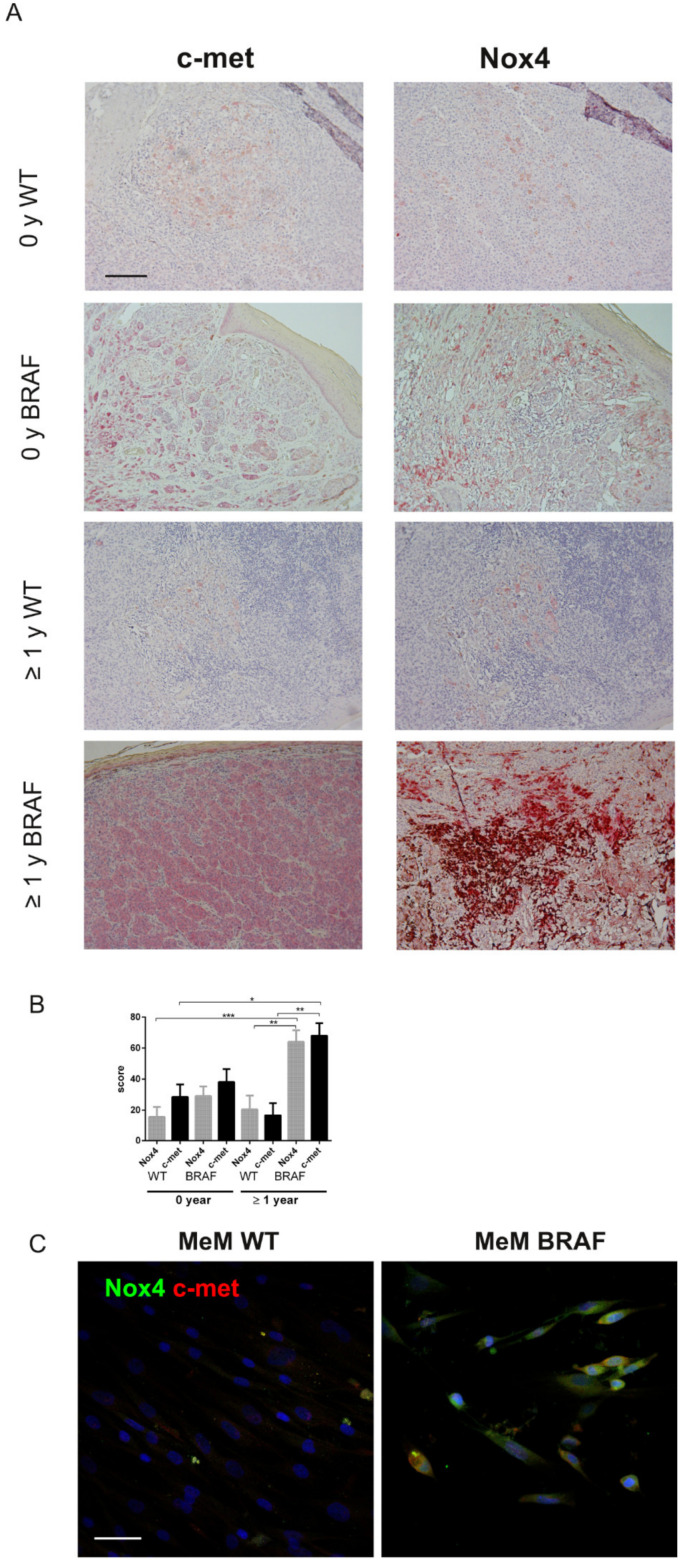
Nox4 and c-met expression in BRAF mutated primary melanoma. (**A**) Representative images of Nox4 and c-met IHC staining (red staining) in primary MeM WT or BRAF mutated. MeM at the time of the diagnosis (0 y) or that developed metastasis after at least 1 year (≥1 y) are shown. Scale bar = 500 µm. (**B**) Percentage of positive cells analyzed by 3 operators are shown in the graphs. * *p-*value < 0.05; ** *p-*value < 0.01; *** *p-*value < 0.001. (**C**) Representative images with DAPI (blue), Nox4 (green) and c-met (red) signals of MeM WT or BRAF mut primary cells. Scale bar = 20 µm.

The images of IHC shown in Figure 2A are representative of the MeM group: all the shown samples are of lesions without ulcerations and with epithelioid cells. The positivity in Nox4 and c-met staining dramatically emerges in BRAF mutated patients in which metastasis occurred after at least 1 year (late metastatic). Statistical evaluation of the positivity scores, shown in the graph of Figure 2B, definitely demonstrated a difference in late metastatic patients between BRAF mut compared to the WT in the IHC picture of Nox4 and c-met.

Furthermore, analysis on cells of primary culture melanoma tissues obtained from a BRAF mutated MeM patient and from a WT patient confirmed the maintenance in culture of both Nox4 and c-met positivity in the BRAF mut MeM sample, as shown in immunofluorescence images of Figure 2C.

### 2.3. Is the Expression of Nox4 Related to c-met Activation?

**Figure 3:**
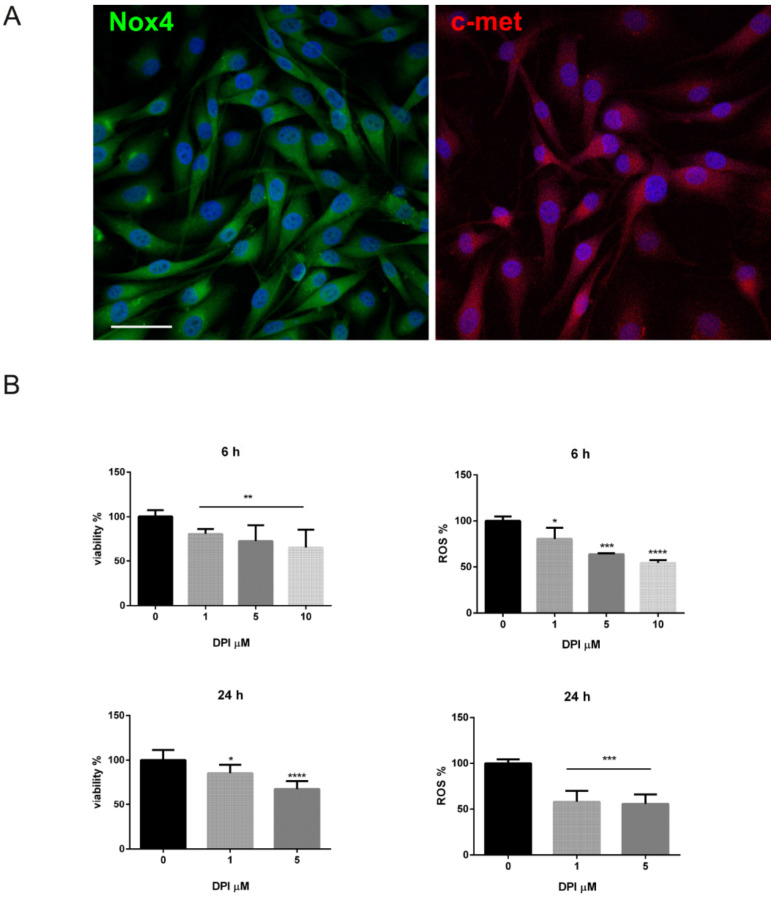
Nox4 and c-met expression in SK-MEL-28 BRAF mutated melanoma cell line. (**A**) Representative images with DAPI (blue), Nox4 (green) or c-met (red) signals of SK-MEL-28. Scale bar = 20 µm. (**B**) Graphs showing MTT assay and ROS levels measured in SK-MEL-28 cells treated with DPI up to 10 µM for 6 or 24 h. * *p-*value < 0.05; ** *p-*value < 0.01; *** *p-*value < 0.001; **** *p-*value < 0.0001.

In order to investigate the interplay between c-met and Nox4, we set up in vitro experiments with a melanoma cell line presenting BRAFV600E mutation, SK-MEL-28. Actually, Nox4 and c-met were expressed in this cell line as well (Figure 3A).

With the purpose to modulate Nox4 activity, we tested the efficacy of diphenyleneiodonium (DPI), a Nox inhibitor [11], on viability and ROS level regulation. Figure 3B shows that DPI, already at 1 µM concentration, is able to decrease intracellular ROS and also MTT absorbance, as viability test, after a short (6 h) exposure, but it is maintained after a long one (24 h). Basing on these observations, 1 µM DPI for 6 h was the experimental condition chosen for the following analyses.

**Figure 4:**
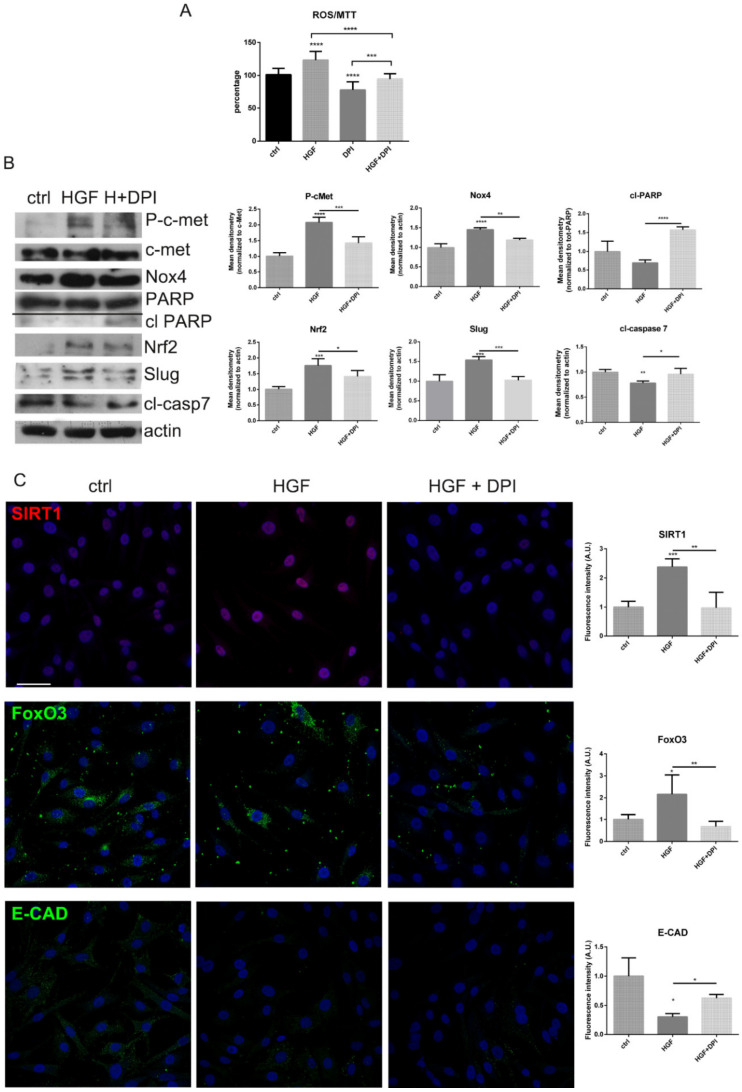
Nox4 and c-met modulation in SK-MEL-28 cells. (**A**) Graph showing ROS levels normalized to MTT values of measured in SK-MEL-28 cells treated with DPI up to 1 µM for 6 h in the presence or absence of HGF. *** *p-*value < 0.001; **** *p-*value < 0.0001. (**B**) Western blot analysis of total lysate of SK-MEL-28 cells treated or not with DPI in the presence of HGF, then revealed with anti-*p*-c-met, anti-c-met, anti- PARP, anti-Nox4, anti-Nrf2, anti-Slug, anti-cleaved caspase 7, and anti-actin as a loading control. The graphs represent the mean ± SD of densitometric analysis of three experiments, normalized to actin or c-met or total PARP values. * *p-*value < 0.05; ** *p-*value < 0.01; *** *p-*value < 0.001; **** *p-*value < 0.0001. (**C**) Representative images with DAPI (blue), FoxO3 or E-CAD (green) or SIRT1 (red) signals of SK-MEL-28 treated as previously reported. Scale bar = 20 µm. Fluorescence intensity of cells visualized in 5 fields for each condition are shown in the graphs. * *p*-value < 0.05; ** *p-*value < 0.01; *** *p-*value < 0.001.

Activation of c-met axis was obtained by the exposure to HGF: indeed, the phosphorylation of the receptor occurred, as shown in WB image of Figure 4B. Meanwhile, HGF interaction with its receptor, did not modify the c-met itself expression, but induced a rise in ROS level (Figure 4A) parallel to an increase on Nox4 presence (Figure 4B). Interestingly, the treatment with DPI significantly counteracted the oxidative stress induced by HGF (Figure 4A). The markers of apoptosis, such as cleaved PARP and casapase 7, were induced by DPI, compared to HGF treated cells (Figure 4B), confirming the viability decrease observed in Figure 3.

The analysis of redox sensitive factors, such as Nrf2 [12], FoxO3 [13] and SIRT1 [14], showed that the presence of HGF, inducing a ROS rise, stimulated a cell defense response through the involvement of such redox related factors (Figure 4B,C). On the other hand, the exposure to DPI, inhibiting the ROS production, avoided this involvement.

Markers of EMT are the increase of Slug [15] and a decline of E-cadherin [16] expression. Notably, the presence of HGF promoted Slug expression (Figure 4B) and affected the staining of E-cadherin (E-CAD) (Figure 4C), but the co-treatment with DPI counteracted these effects.

**Figure 5:**
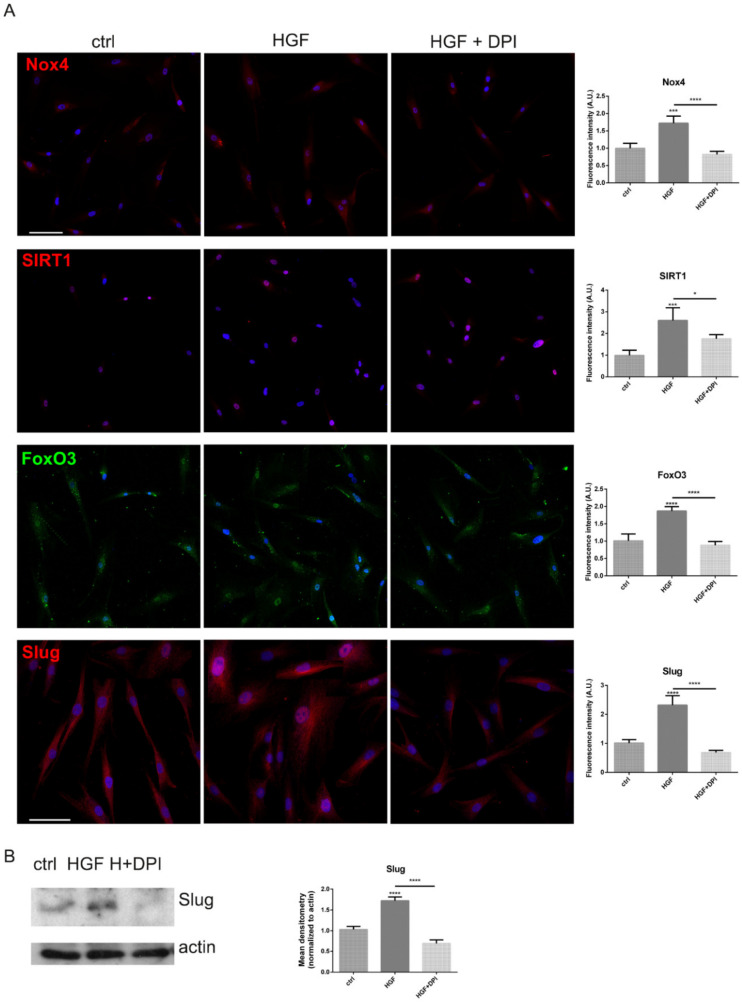
Nox4 and c-met modulation in MeM BRAF mutated primary melanoma cells. (**A**) Representative images with DAPI (blue), FoxO3 (green) or Nox4, SIRT1, Slug (red) signals of BRAF mutated primary cells treated as previously reported. Scale bar = 20 µm. Fluorescence intensity of cells visualized in 5 fields for each condition are shown in the graphs. * *p*-value < 0.05, *** *p-*value < 0.001. (**B**) Western blot analysis of total lysate of MeM BRAF mut primary cells treated or not with DPI in the presence of HGF, then revealed with anti-Slug and anti-actin as a loading control. The graph represents the mean ± SD of densitometric analysis of three experiments, normalized to actin values. **** *p-*value < 0.0001.

More importantly, these observations were confirmed in primary melanoma cells. Testing the efficacy of DPI in primary melanoma culture derived from a BRAF mut MeM (already shown in Figure 2C), a similar effect occurred: indeed, the increase in Nox4, FoxO3, SIRT1 and Slug presence, induced by HGF, was avoided by DPI exposure, as reported in Figure 5A,B.

## 3. Discussion

Altered redox metabolism and antioxidant production are common features among melanomas, and that regulation of redox enzymes may be a viable treatment strategy for melanomas in general. Here, we showed that in cells overexpressing c-met, the HGF receptor, an up-regulation of a ROS-generating enzyme, NADPH oxidase 4 (Nox4), occurred. The pivotal role of Nox4 in melanoma, that this observation suggests, is consistent with data discussed by Meitzler in 2017 [9] where it has been reported that most melanomas showed diffuse positivity of Nox4, even if in a different cellular distribution. However, we noticed a huge expression of both c-met and Nox4 in metastatic melanoma, unlike in cases without recurrences. Actually, in patients showing lymph nodes positivity, the presence of c-met and Nox-4 starts to increase in BRAF mutated cases, but becomes statistically relevant only in cases of distant metastasis, indicating Nox4 as a possible melanoma severity marker as well as c-met. This observation confirms a link between HGF/c-met pathway and Nox4 activation and suggests a role of this interplay in the progression of melanoma. Indeed, this connection has been reported in other tissues: Usatyuk et al. demonstrated a significant role for HGF-induced c-Met/PI3k/Akt signaling and NADPH oxidase activation in lamellipodia formation and motility of lung endothelial cells [17]. They showed that HGF stimulated translocation of Nox subunits, namely p47(phox)/Cortactin/Rac1, to lamellipodia and ROS generation. Moreover, intracellular ROS generated by the NADPH oxidase, most likely Nox4, transmits cell survival signals on melanoma cells maintaining cell viability [18]. These considerations already highlight the hypothesis of a treatment of melanoma with Nox inhibitors. This suggestion is even more strengthened for some patients: indeed, here we showed that there is a tremendous difference in the expression of both these proteins comparing BRAF mutated cases not yet presenting metastasis (but that will be) and WT. In BRAF mutated samples we observed the highest and more diffused staining for both these markers. On the other hand, between WT and BRAF mutated, the labeling was similar in samples where the metastatic process was already started. Notably, HGF/c-met pathway activation triggers both innate and acquired resistance to BRAF inhibitors, thus affecting this pathway, that involves ROS production, could be useful to contrast such drug resistance.

Therefore, we employed a Nox inhibitor, DPI, to test its efficacy on the modulation of viability, redox regulation and metastasis process occurrence. In vitro experiments with a BRAF mutated MeM cell line demonstrated that DPI is able to induce apoptosis and to counteract the effects due to the activation of the HGF/c-met pathway, including the first step, the c-met phosphorylation. Actually, ROS affects many phosphatases [19], therefore a decrease of ROS, due to DPI, could induce a decline in c-met phosphorylation. Interestingly, the exposure to HGF triggered ROS production by raising Nox4 presence, while DPI co-treatment avoided this effect, may be both inhibiting c-met and Nox4 activation.

Downstream steps, such as the increase of SIRT1, Nrf2, FoxO3A, redox dependent molecules, as defense answer to the cell stress caused by HGF-derived ROS, were reduced in the presence of DPI. During EMT, process that can explain the invasiveness and aggressiveness of these tumors which metastasize, epithelial cells lose expression of the adhesion protein E-cadherin in favor of N-cadherin. It has been reported in melanoma that SIRT1, a nicotinamide adenine dinucleotide (NAD+)-dependent protein deacetylase, induces EMT by accelerating E-cadherin degradation and facilitates melanoma metastasis [20]. Our data confirmed that SIRT1 is stimulated by HGF exposure, but inhibited by DPI presence.

Oxidative stress activates SIRT1 that modulates p53 and FoxO transcription factors. FOXO causes in turn the activation of free radical scavenging genes to protect against oxidative stress [21]. The positive modulation of FoxO3 that we noticed in the presence of HGF can be the effect of ROS increase, and DPI, avoiding this ROS rise, prevents the FoxO3 activation.

Another transcription factor usually involved in redox unbalance is Nrf2 that is main orchestrators of the cellular antioxidant response. Moreover, it has been demonstrated that in cancer cell lines even Nrf2 promotes EMT by downregulation of E-cadherin expression through unknown mechanisms [22]. DPI was able to avoid Nrf2 increase as well.

Furthermore, here we showed that the EMT marker Slug fell with the DPI treatment that even induced a slight rise in E-cadherin expression, suggesting a regulation of tumor invasiveness.

Data obtained in SK-MEL-28 cell line are consistent with the one obtained in primary BRAF mutated melanoma cells, thus highlighting that the use of a Nox4 inhibitor could potentiate the current therapeutic strategy to treat melanoma patients with BRAF mutations.

## 4. Materials and Methods

### 4.1. Study Population

We retrospectively analyzed samples from 60 consecutive patients with primary melanoma retrieved from the dermatology and pathology database, according to local ethical guidelines. The inclusion criteria of our retrospective study were (1) the presence of a complete set of histopathological analysis, (2) availability of histopathological sections and (3) clinical records of patients’ follow-up for at least 4 years from the diagnosis (4) not to be included in other studies. Among these melanomas, 41 were metastasizing (MeM samples) and 19 non-metastatic (NoM samples), but matched by anatomical site of the primary tumor and year of diagnosis. In the MeM group, 16 patients presented distant metastasis after at least 1 year, and 25 at the time of melanoma diagnosis: among these, a subgroup of 9 patients had only positive lymph nodes (MeL). Around half of all the patients had BRAF600V mutation.

The study was approved the 01/06/2018 by the Area Vasta Emilia Nord committee (protocol number 175/2018).

### 4.2. Histological Study

Histopathology evaluation and IHC analysis were performed at the pathology department. Immunohistochemistry was performed on formalin-fixed, paraffin-embedded melanoma sections (4 µm thick), using an automated system according to the manufacturer’s protocol (Ventana Medical Systems, Tucson, AZ, USA). c-met (Ventana Medical Systems, Tucson, AZ, USA), Nox4 (Santa Cruz, CA, USA) primary antibodies were diluted to 1:50 in antibody dilution buffer and incubated with tissue samples overnight at 4 °C. Primary antibodies were developed with the use of ultraview Universal Alkaline Phosphatase Red Detection Kit (Ventana Medical Systems, Tucson, AZ, USA.). Slides were counterstained with haematoxylin. For all these procedures, staining without the primary antibody was carried out as a negative control. IHC positivity of all human melanoma specimens was performed independently by three investigators (T.M., M.Z. and F.B.). IHC scoring: to score tumor section for each target antigen, three different representative 400× magnification fields of at least 100 tumor cells were taken. Total cells and cells with positive nuclear staining were counted and the percent positive cells in each high-power field (HPF) were calculated.

### 4.3. Cell Culture and Treatments

SK-MEL-28 cells, derived from primary malignant melanoma cells BRAF mutated, (American Type Culture Collection, Manassas, VA, USA) were used and cultured in DMEM-F12 (Sigma Aldrich, St Louis, MO, USA) enriched with 10% fetal bovine serum (FBS), penicillin 100 U/mL, streptomycin 100 µg/mL and 2 mM L-glutamine. The cells were maintained at 37 °C and 5% CO_2_.

The use of melanoma biopsies was approved by Ethical Review Committee of the Modena Hospital (protocol number 1338/CE; 4/09). Biopsies were taken from the Dermatology Unit of Modena Hospital only in patients eligible for the study, after gave inform consent.

Primary melanoma cells derive from a BRAF mutated nodular tumor and from a WT MeM sample. Immediately after surgical resection, melanoma cells were dissociated into single cells with collagenase I (1 mg/mL; Biochem, Nuoro, Italy) as previously indicated [23]. The single cell suspension was filtered by 100 µm strainer and single cells were harvested. Cells were maintained in DMEM-F12 (Sigma Aldrich, St Louis, MO, USA), 10% heat-inactivated FBS, penicillin 100U/mL, streptomycin 100 µg/mL and 2 mM L-glutamine.

### 4.4. MTT Assay

SK-MEL-28 were seeded in 96-well plates in 100 μL of a culture medium, 4 replicates for each condition, at the density of 500 cells/well. Cells were then treated with the Nox inhibitor DPI for 6 to 24 h. HGF 1 ng/mL (Sigma Aldrich, St Louis, MO, USA) treatment was performed during the DPI exposure for 6 h at the concentration of 1 µM. At the end of experiment, 0.5 mg/mL MTT was added and incubated for 3 h at 37 °C. After incubation, the medium was removed and acidified isopropanol was added to solubilize the formazan salts [24]. The absorbance was measured at 570 nm using a microplate spectrophotometer (Appliskan, Thermo-Fisher Scientific, Vantaa, Finland).

### 4.5. ROS Detection

To evaluate intracellular ROS levels, dichlorodihydrofluorescein diacetate (DCFH-DA) assay was performed similarly to as previously described [25]. After cell treatments, cell culture medium was removed, and the 5 μM DCFH-DA was incubated in PBS for 30 min, at 37 °C and 5% CO_2_. The cell culture plate was washed with PBS, and fluorescence of the cells was read at 485 nm (excitation) and 535 nm (emission) using the multiwall reader Appliskan (Thermo-Fisher Scientific, Vantaa, Finland). Cellular autofluorescence was subtracted as a background using the values of the wells not incubated with the probe.

### 4.6. Cellular Extracts Preparation

Cell extracts were obtained as previously described [26]. Briefly, cells were treated with lysis buffer (20 mM Tris-Cl, pH 7.0; 1% Nonidet P-40; 150 mM NaCl; 10% glycerol; 10 mM EDTA; 20 mM NaF; 5 mM sodium pyrophosphate; and 1 mM Na_3_VO_4_) and freshly added Protease Inhibitor Cocktail (Sigma Aldrich) and para-nitrophenylphosphate (Sigma Aldrich) at 4 °C for 20 min. Lysates were sonicated, cleared by centrifugation and immediately boiled in SDS reducing sample buffer.

### 4.7. SDS PAGE and Western Blot

Whole cell lysates from SK-MEL-28 and primary MeM cells were processed as previously described [26]. Primary antibodies were raised against the following molecules: Actin and Nox4 (Sigma-Aldrich, St Louis, MO, USA), phospho c-met, c-met and cleaved caspase 7 (Cell Signaling Technology, Lieden, Netherlands), Nrf2 and Slug (Abcam, Cambridge, UK), PARP (Santa Cruz Biotechnology, Santa Cruz, CA, USA).

Secondary antibodies, used at 1:3000 dilutions, were all from Thermo Fisher Scientific (Waltham, MA, USA).

### 4.8. Immunofluorescence and Confocal Microscopy

For immunofluorescence analysis, SK-MEL-28 and primary MeM cells seeded on coated coverslips were processed and confocal imaging was performed using a Nikon A1 confocal laser scanning microscope, as previously described [27].

Primary antibodies to detect Sirt1, c-met (Cell Signaling Technology, Lieden, Netherlands), E-cad (Abcam, Cambridge, UK), Nox4 and FoxO3 (Santa Cruz Biotechnology, Santa Cruz, CA, USA) were used following datasheet recommended dilutions. Alexa secondary antibodies (Thermo Fisher Scientific, Waltham, MA, USA) were used at 1:200 dilutions.

The confocal serial sections were processed with ImageJ software to obtain three-dimensional projections. The image rendering was performed by Adobe Photoshop software.

The cell fluorescence signal was quantified using ImageJ and applying the following formula:

Corrected Total Cell Fluorescence (CTCF) = Integrated Density − (Area of selected cell × Mean fluorescence of background readings).

### 4.9. Statistical Analysis

Experiments were performed in triplicate. For quantitative comparisons, values were reported as mean ± SD based on triplicate analysis for each sample. To test the significance of observed differences among the study groups, one-way ANOVA with Bonferroni post hoc test or Student’s t test were applied. A *p-*value < 0.05 was considered to be statistically significant. Statistical analysis and plot layout were obtained by using GraphPad Prism^®^ release 6.0 software (www.graphpad.com).

## Figures and Tables

**Table 1:** Clinical data of melanoma samples—mean age values ± SD, median values with range of Breslow thickness and anatomic site of the lesions (T = trunk, HN = head and neck, UL = upper limb, LL = lower limb).

M Type	Age (Years)	Breslow (mm)	Anatomic Site
NoM	53.2 ± 13.2	1.1 (range 0.0 ≥ 4.0)	10T, 6HN, 1UL, 2LL
MeL	65.8 ± 12.4	3.2 (range 1.1 ≥ 8.0)	4T, 2HN,1LL
MeM	60.1 ± 16.3	3.6 (range 1.0 ≥ 50.0)	20T, 4HN, 3UL, 5LL

## Data Availability

Not applicable.

## Abbreviations

BSA	bovine serum albumin
DCFH-DA	dichlorodihydrofluorescein diacetate
DAPI	4’,6-diamidino-2-phenylindole
DPI	diphenyleneiodonium
EDTA	ethylenediaminetetraacetic acid
EMT	epithelial-mesenchymal transition
HGF	hepatocyte growth factor
IHC	immunohistochemical
MeM	metastasizing melanoma
MeL	melanoma with positive lymph nodes
Nox	NADPH oxidase
NoM	non-metastatic melanoma
PBS	phosphate buffered saline
ROS	reactive oxygen species
SDS	sodium dodecyl sulphate
WB	western blot
